# Soil Biochemical Responses to Nitrogen Addition in a Bamboo Forest

**DOI:** 10.1371/journal.pone.0102315

**Published:** 2014-07-16

**Authors:** Li-hua Tu, Gang Chen, Yong Peng, Hong-ling Hu, Ting-xing Hu, Jian Zhang, Xian-wei Li, Li Liu, Yi Tang

**Affiliations:** 1 College of Forestry, Sichuan Agricultural University, Ya'an, Sichuan, China; 2 Personnel Department, Sichuan Agricultural University, Ya'an, Sichuan, China; 3 College of Horticulture, Sichuan Agricultural University, Ya'an, Sichuan, China; Tennessee State University, United States of America

## Abstract

Many vital ecosystem processes take place in the soils and are greatly affected by the increasing active nitrogen (N) deposition observed globally. Nitrogen deposition generally affects ecosystem processes through the changes in soil biochemical properties such as soil nutrient availability, microbial properties and enzyme activities. In order to evaluate the soil biochemical responses to elevated atmospheric N deposition in bamboo forest ecosystems, a two-year field N addition experiment in a hybrid bamboo (*Bambusa pervariabilis* × *Dendrocalamopsis daii*) plantation was conducted. Four levels of N treatment were applied: (1) control (CK, without N added), (2) low-nitrogen (LN, 50 kg N ha^−1^ year^−1^), (3) medium-nitrogen (MN, 150 kg N ha^−1^ year^−1^), and (4) high-nitrogen (HN, 300 kg N ha^−1^ year^−1^). Results indicated that N addition significantly increased the concentrations of NH_4_^+^, NO_3_^−^, microbial biomass carbon, microbial biomass N, the rates of nitrification and denitrification; significantly decreased soil pH and the concentration of available phosphorus, and had no effect on the total organic carbon and total N concentration in the 0–20 cm soil depth. Nitrogen addition significantly stimulated activities of hydrolytic enzyme that acquiring N (urease) and phosphorus (acid phosphatase) and depressed the oxidative enzymes (phenol oxidase, peroxidase and catalase) activities. Results suggest that (1) this bamboo forest ecosystem is moving towards being limited by P or co-limited by P under elevated N deposition, (2) the expected progressive increases in N deposition may have a potential important effect on forest litter decomposition due to the interaction of inorganic N and oxidative enzyme activities, in such bamboo forests under high levels of ambient N deposition.

## Introduction

Anthropogenic nitrogen (N) deposition, primarily from food and energy production [1], has increased three- to five-fold over the last century [2], and presently adds more than 200 Tg yr^−1^ of N to terrestrial and ocean ecosystems [1]. It is predicted that N deposition will increase considerably in the 21^st^ century with the largest increases occurring in East and South Asia [3]. Studies have shown that the increased N deposition substantially affected a range of essential processes relevant to the carbon (C) cycle in terrestrial ecosystems [4], [5].

Carbon sequestration in forests depends on the balance between C fixation through plant growth and C loss through decomposition of soil organic matter (SOM) and plant litter. The response of plant growth in forests is generally positive [6]–[8], since the primary productivity in most forests is N limited [5]. While, the effects of N deposition on SOM and plant litter decomposition are highly controversial [9]–[13] due to the variability of forest properties and environmental factors. Soil extracellular enzymes (SEE) produced by microbes and plant roots were direct executors in the decomposition of litter and soil organic matter (SOM) [14]. Soil extracellular enzymes have two functions: to decompose complex substances into simple molecules and to supply sources to SEE producers [15]. Microbes and plants can never gain sources from complicated organism and the C and nutrient cycles would end without SEE [14]. Previous studies found that the response of SEE to elevated N inputs can explain the effects of exogenous N on the decomposition of SOM and litter [16]. In these studies, the best explanation for changes in decomposition rates following N deposition or N addition is the effect of N on the activity of the lignin-degrading enzyme phenol oxidase [16], [17]. In general, the activities of cellulose-degrading enzymes would be stimulated after N addition, while lignin-degrading enzymes would be inhibited [18]. The response of SEE activities to N deposition in different ecosystems varied because of the diversity in nutrient availability [19], lignin content in litter [16], and the C/N ratio of litter and soil [16].

Carbon pools in plants and soils are closely linked through the nutrient cycle in forests. Thus, N deposition generally indirectly affects ecosystem processes, such as plant growth, litter decomposition and soil respiration, through the changes in soil biochemical properties such as soil nutrient availability, microbial properties and enzyme activities. Over the last decade, a considerable number of studies have demonstrated that experimental N addition has pronounced effects on soil available nutrients, soil microbial activities and structures, and soil enzyme activities [16], [17], [18], [20]–[22]. However, ecosystem responses differed among ecosystem types.

It should be noted that most studies regarding the effects of N addition on soil properties have been conducted in coniferous and in broad-leaved forests. Bamboo forests are one of the most important forest types in the world [23]. China is one of the distribution centers of bamboo, and China's bamboo forests account for 15.4% (4.84 million ha in 2005) of the total area of bamboo worldwide [23]. Bamboo forests contribute about 10% of the C stocks in the living biomass of forests in China [24]. Therefore, bamboo forests play an important role in regional and global C cycling. Furthermore, bamboo forests are mainly distributed in the southern provinces of China. At present, southern China is experiencing rates of N deposition that are well above the global average [25]–[27]. The largest increases of N deposition in the world are projected to occur in this region over the next few decades [3]. Therefore, understanding the effect of increasing N deposition on the soil in bamboo forests is critical for predicting how the ecosystem processes regarding C cycling and nutrient circulation in bamboo ecosystems will respond to human activities.

To evaluate the initial soil biochemical responses to elevated atmospheric N deposition, we conducted experimental N addition treatments in a hybrid bamboo (*Bambusa pervariabilis × Dendrocalamopsis daii*) plantation over a two year period. The aim of this study is to examine the impacts of N addition on soil acidity, nutrient availability, microbial properties and soil enzyme activities.

## Materials and Methods

### Site description

The simulated N deposition experiment was conducted in a *Bambusa pervariabilis × Dendrocalamopsis daii* (hybrid bamboo) (10 ha) stand in Liujiang, Sichuan, China (29°42′25″ N, 103°14′38″ E, altitude 600 m above sea level). This region experiences an elevation-modified humid subtropical climate. The annual mean relative humidity was 86%. The mean temperature and annual precipitation in 2007, 2008, and 2009 were 16.8, 15.8, and 18.3°C, and 1758, 1847 and 1984 mm, respectively. The background wet N deposition measured in 2008 and 2009 was 82 and 113 kg N ha^−1^ year^−1^, respectively [27], [28]. The total atmospheric N deposition (N deposition in throughfall and stemflow in a bamboo forest) in Liujiang was approximately 131.5 kg N ha^−1^ year^−1^ in 2009, which was one of the highest in the world [27]. The site was planted on former cropland in 2000 as part of the National Project of Converting Farmland to Forests (NPCFF). The site was close to the *Pleioblastus amurus* site (about 1 km apart from each other) used for the already- reported studies on carbon sequestration [29] and soil respiration [30] under N addition experiments. At the time of our study, the plant density was 13,320 stems ha^−1^ and the mean diameter at breast height (DBH) was 6 cm. The surface soil horizon (0–10 cm) contained 13.4 mg g^−1^ C and 1.58 mg g^−1^ N. The average soil depth to bedrock was approximately 1 m, and the thickness of the surface organic layer was approximately 1 cm before the experimental treatments began. The soil at the site is classified as a Dystric Purpli-Orthic Primosol (Lithic Dystrudepts according to USDA Soil Taxonomy) derived from purple sandstone and shale. There was very little shrub or herb in the understory at the time of the experiment.

The research site is owned by Sichuan Agricultural University. The field studies did not involve endangered or protected species and no specific permits were required for the described field studies.

### Microclimate and litterfall measurements

Air temperature and precipitation were measured using a Davis Weather Station (Vantage Pro, Davis Inc. USA) located adjacent to the experimental area. Litterfall was collected in ten 50×50 cm nylon mesh traps placed randomly in the experimental site but outside of the treated plots. Soil temperature was measured on each sampling date using thermometer.

### Experimental treatments

Nitrogen addition experiments were initiated in January 2008. Twelve plots were established and divided into four N addition treatments: control (CK; without N added), low-N (LN; 50 kg N ha^−1^ year^−1^), medium-N (MN; 150 kg N ha^−1^ year^−1^), and high-N (HN; 300 kg N ha^−1^ year^−1^), with three replicates each. The plots measured 3×3 m, spaced at approximately about 5 m intervals, and randomly selected to receive treatments. The addition of fertilizer (NH_4_NO_3_) occurred monthly in twelve equal applications beginning in January 2008. During each application, the fertilizer was weighed, dissolved in 1 L of water, and applied to each plot using a portable sprayer. The control plot received 1 L of water without fertilizer.

### Soil sample collection and analysis

From January to December 2009, five subsamples of surface mineral soil (0–10 cm) were monthly taken from each plot using a 27 mm diameter soil auger. Subsamples of each plot were mixed to form a composite sample and the visible roots were removed by tweezers. The soil sample was homogenized, passed through a 2 mm sieve and stored at 4°C for analysis within one week. The soil water content was determined by dried at 105°C using a subsample (10 g) from each plot. For determining soil total organic carbon (TOC) and total nitrogen (TN), air-dried subsamples were ground to sieve through the mesh with size of 0.25 mm.

The pH value was determined by a glass electrode in aqueous extracts. Soil TOC was measured by the dichromate digestion method [31]. Soil TN was determined through acid digestion, using the Kjeldahl method [32]. Available N (ammonium and nitrate) was extracted with a 2 M KCl solution, and measured colorimetrically. Soil microbial biomass C (MBC) and soil microbial biomass N (MBN) were measured using the chloroform fumigation extraction technique [33], [34], [35] by a total CN analyzer (Shimadzu model TOC-V_cPH+TNM-1_, Kyoto, Japan). The Bray-2 method [36] was used to measure soil available P.

Invertase (β-D-fructofuranoside fructohydrolase, EC 3.2.1.26; abbreviation: βF) activity was measured with the Frankenberger Jr and Johanson method [37]. Urease (EC 3.5.1.5) activity was measured spectrophotometrically (610 nm) according to Sinsabaugh et al. [38]. The activity of acid phosphatase (AcPh; EC 3.2.1.2) was determined following published protocols [20] using 4-methyumbelliferyl (MUB) phosphate as a substrate. Phenol oxidase (PhOx; EC 1.10.3.2) and peroxidase (POD; EC 1.11.1.7) activities were measured spectrophotometrically using *L*-3, 4-dihydroxyphenylalanine (DOPA) as the substrate [20]. Catalase (CAT; EC 1.11.1.6) activity was determined with the Cohen et al. [39] method where decomposed hydrogen peroxide is measured by its reaction to excess potassium tetraoxomanganate (VII). Enzyme activity was calculated as the µmoles of substrate converted per hour (or per minute for CAT) for each gram of dried sample.

In February, April, June, August, October and December 2009, three undisturbed soil cores (100 cm^3^) (0–10 cm soil horizon) were collected using the accessories of barometric process separation (BaPS) system (UMS Inc. Germany) from each plot. The soil cores were returned to the laboratory and stored on ice in coolers until analysis. Gross nitrification and denitrification rates were measured using BaPS system according to Ingwersen et al. [40]. The unit of gross nitrification and denitrification rates is µg N kg^−1^ h^−1^ (based on the dry weight of the soil samples).

### Statistical analyses

All analyses were conducted using SPSS 15.0 for Windows (SPSS Inc. USA). Repeated measures ANOVA with Fisher's LSD tests were performed to examine the soil temperature, moisture, pH, TOC, TN, NH_4_^+^-N, NO_3_^−^-N, MBC, MBN, and activities of *β*F, urease, AcPh, PhOx, POD, CAT, gross nitrification rate, and denitrification rate for the different treatments. Significant effects were determined at *α* = 0.05 unless otherwise stated. Mean values in the text are given ± 1SE.

The accumulative activities of enzymes were calculated by the following equation:
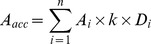



*A_acc_* represents the accumulative activity in one year for 1 kg soil (mol kg^−1^ yr^−1^); *A_i_* is the mean value of enzyme activity in the month (µmol g^−1^ h^−1^); *i* = 1, 2, 3, …, *n* (i.e., *i* = 1 represents January, *i* = 2 represents February); *n* is the number of months in one year (12); *k* is the unit conversion factor (*k* = 0.024 for βF, urease, AcPh, PhOx, POD; *k* = 1.44 for CAT) to converse the unit from µmol g^−1^ h^−1^ to mol kg^−1^ day^−1^, and *D_i_* is the number of days in a month.

## Results

### Microclimate and litterfall

**Figure 1:**
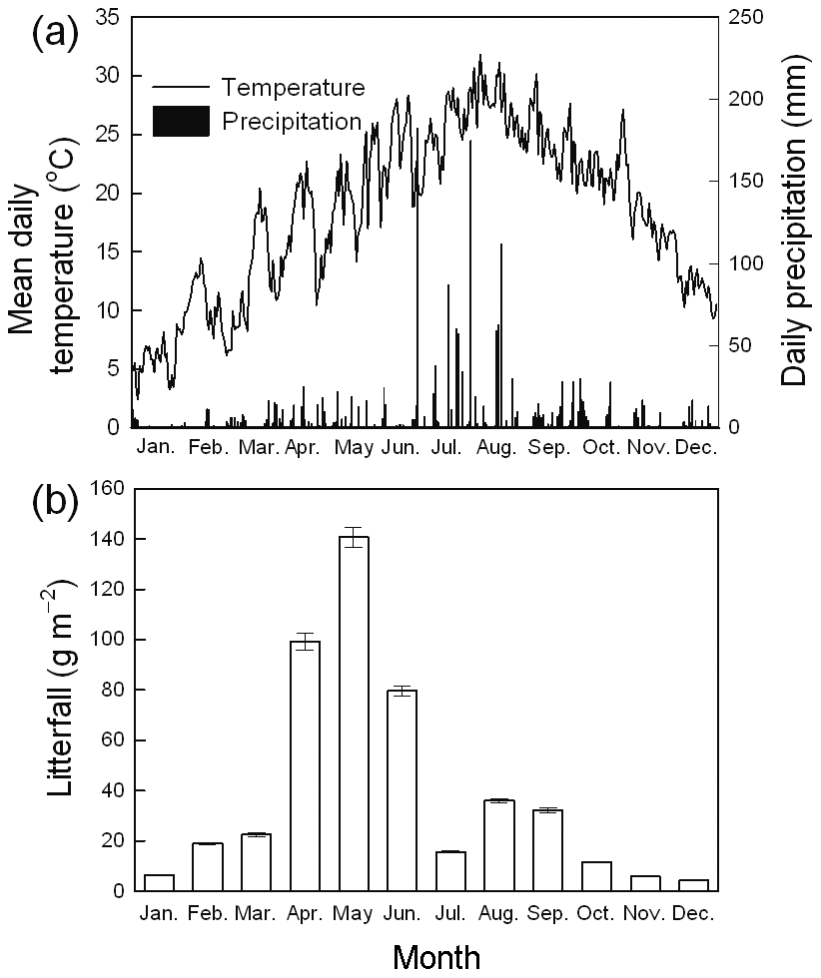
Seasonal variations of air temperature, precipitation and litterfall in a *Bambusa pervariabilis* × *Dendrocalamopsis daii* plantation from January to December 2009. (a) Mean daily temperature and daily precipitation; (b) monthly litterfall. Bars indicate mean ± SE, *n* = 10.

**Figure 2:**
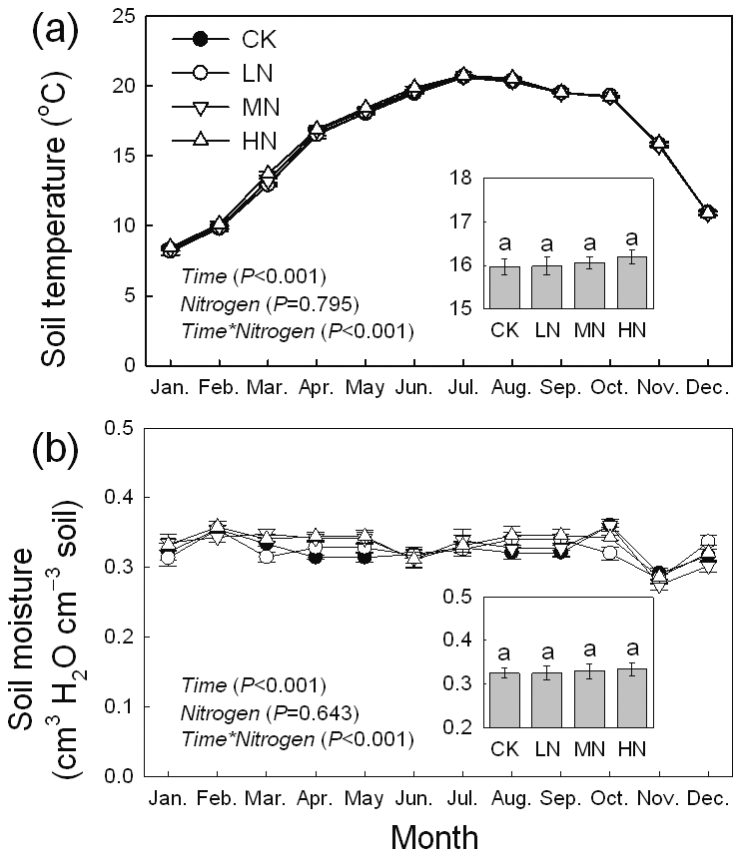
Seasonal variations of soil temperature and soil moisture in a *Bambusa pervariabilis* × *Dendrocalamopsis daii* plantation from January to December 2009 (mean ± SE, *n* = 3). The treatments were: CK (without N added), LN (50 kg N ha^−1^ year^−1^), MN (150 kg N ha^−1^ year^−1^), and HN (300 kg N ha^−1^ year^−1^) with three replicate plots for each treatment. Monthly applications of NH_4_NO_3_began in January 2008. (a) Soil temperature at 10 cm below surface; (b) volumetric soil moisture in the 0–20 cm soil horizon. Average values of yearly measures were exhibited in the histograms (mean ± SE, *n* = 3). Results of repeated measures ANOVAs are shown in text. Different letters indicate significant difference among N addition treatments when nitrogen effect is significant (*P*<0.05, Fisher's least significant difference test).

There were obvious seasonal variations for temperature and precipitation at the experimental site (Figs. 1a,b). Precipitation in the study period (2009) was 1984 mm and the mean annual air temperature was 18.3°C. Mean litterfall in the *P. amarus* plantation was 473±25 g m^2^ year^−1^ over the study period (Fig. 1b). The litterfall mass showed a strong seasonal pattern that peaked in May. The litterfall from April to June accounted for 68% of annual total litterfall. Soil temperature at 10 cm soil depth ranged from 8.3°C in January to 20.6°C in July at the control plots (Fig. 2a). The seasonal variations in soil temperature were similar to air temperature. However, soil moisture was nearly stable, ranging from 0.29 to 0.36 cm^3^ H_2_O cm^−3^ soil (Fig. 2b). There was no significant difference in soil temperature or soil moisture among the plots during the study period (*P* = 0.795 and 0.643, respectively).

### pH, nutrient availability and microbial properties

**Figure 3:**
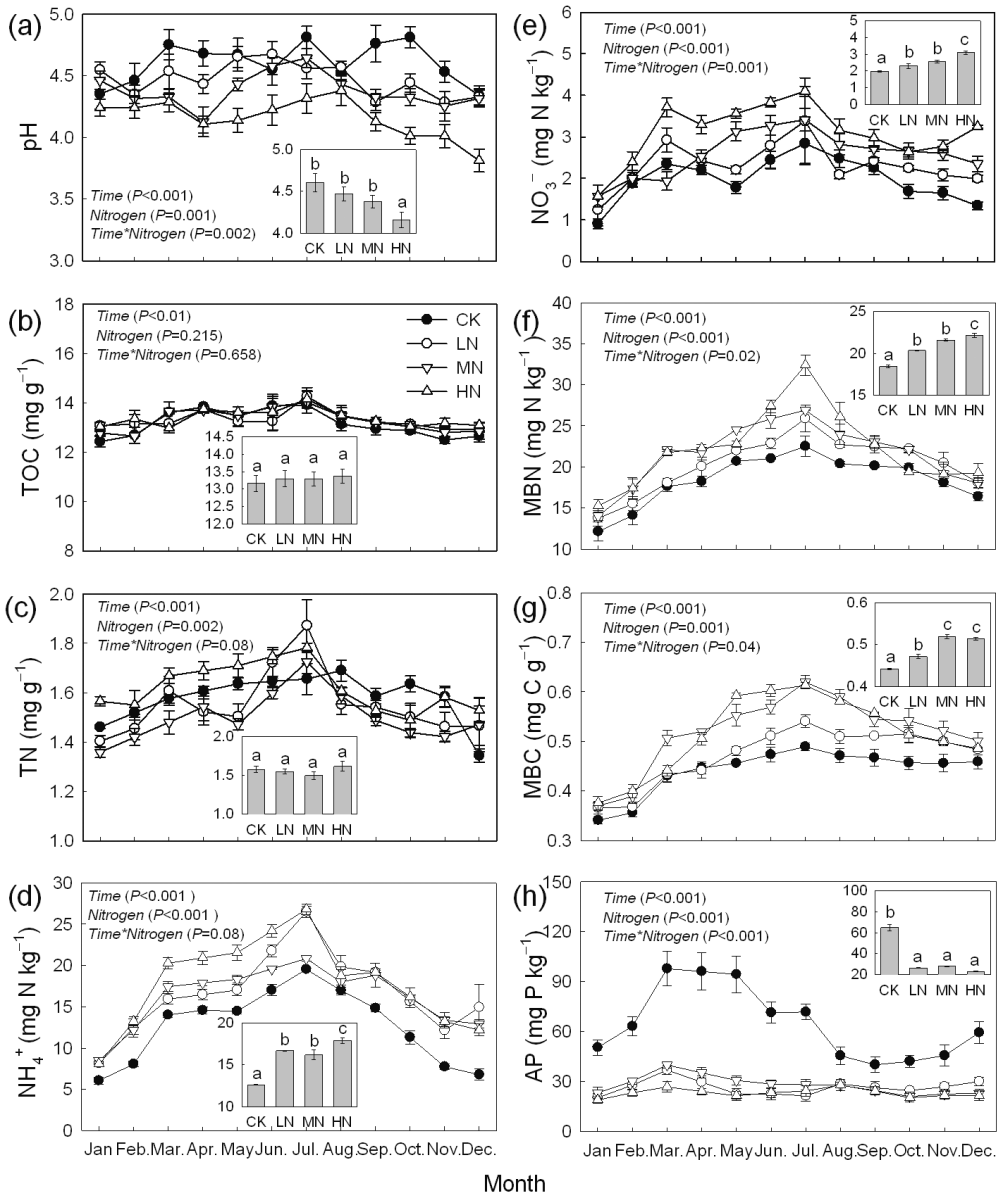
Seasonal variations of soil properties in a *Bambusa pervariabilis* × *Dendrocalamopsis daii* plantation from January to December 2009 (mean ± SE, *n* = 3). TOC: total organic carbon; TN: total nitrogen; MBN: microbial biomass nitrogen; MBC: microbial biomass carbon; AP: available phosphorus. Average values of yearly measures were exhibited in the histograms (mean ± SE, *n* = 3). Results of repeated measures ANOVAs are shown in text. Different letters indicate significant difference among N addition treatments when nitrogen effect is significant (*P*<0.05, Fisher's least significant difference test). CK, LN, MN and HN are as in Fig. 2.

**Figure 4:**
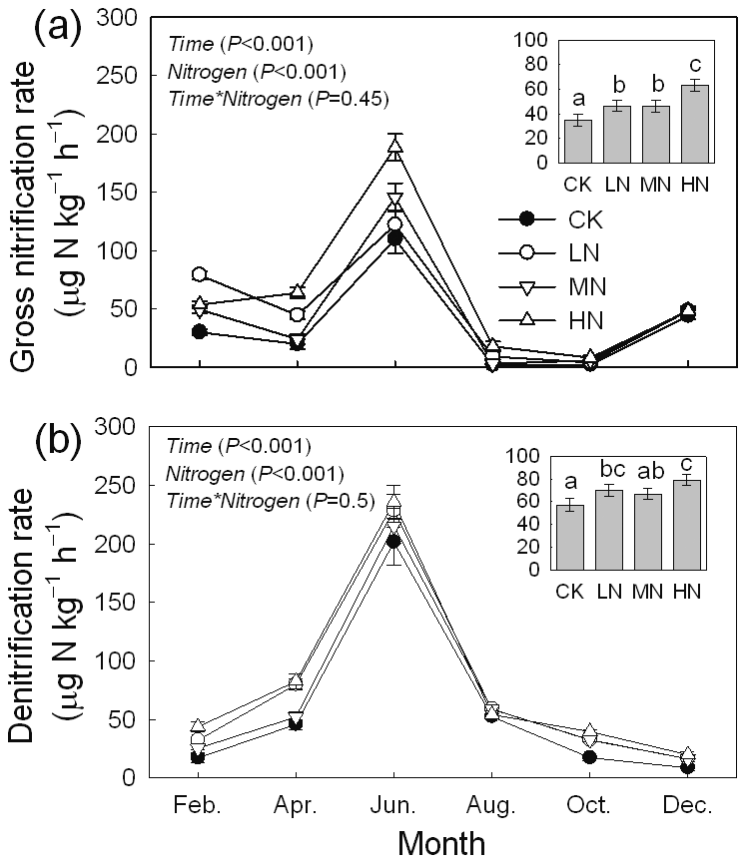
Seasonal variations of soil gross nitrification and denitrificantion rates in a *Bambusa pervariabilis* × *Dendrocalamopsis daii* plantation in 2009 (mean ± SE, *n* = 3). Average values of yearly measures were exhibited in the histograms (mean ± SE, *n* = 3). Results of repeated measures ANOVAs are shown in text. Different letters indicate significant difference among N addition treatments when nitrogen effect is significant (*P*<0.05, Fisher's least significant difference test). CK, LN, MN and HN are as in Fig. 2.

The results of repeated measures ANOVAs indicated that the pH and the concentrations of TOC, TN, NH_4_^+^, NO_3_^−^, MBN, MBC, and AP exhibited significant seasonal patterns (*P*<0.001, Fig. 3). Overall, the concentrations of TN, NH_4_^+^, NO_3_^−^, MBN and MBC peaked in July, and AP peaked in March. The pH values in CK, LN, MN and HN were 4.60±0.11, 4.47±0.08, 4.38±0.08 and 4.16±0.09, respectively, and the pH in all the three treatments were significantly decreased (Fig. 3a). There was no significantly difference between treatments in TOC concentration (Fig. 3b). The addition of N did not significantly affect soil TN concentrations (Fig. 3c). The concentrations of NH_4_^+^, NO_3_^−^, MBN, and MBC increased significantly under all three N addition treatments (Figs. 3d, e, f, g). The concentration of AP decreased 57% to 64% after N added, and the differences between the control and all the N treatments were significant (Fig. 3h). Soil gross nitrification and denitrification rates exhibited significant seasonal variations (*P*<0.001), and both rates peaked in June (Fig. 4). The average gross nitrification rates for CK, LN, MN, and HN were 34.6±4.6, 46.2±4.5, 46.0±4.7, and 63.2±4.7 µg N kg^−1^ h^−1^, respectively. The mean rates of denitrification were 57.0±5.7, 69.6±5.3, 66.7±4.8 and 79.2±5.0 µg N kg^−1^ h^−1^ in CK, LN, MN, and HN plots, respectively. The addition of N significantly increased the nitrification and denitrification rates.

### Soil enzyme activities

**Figure 5:**
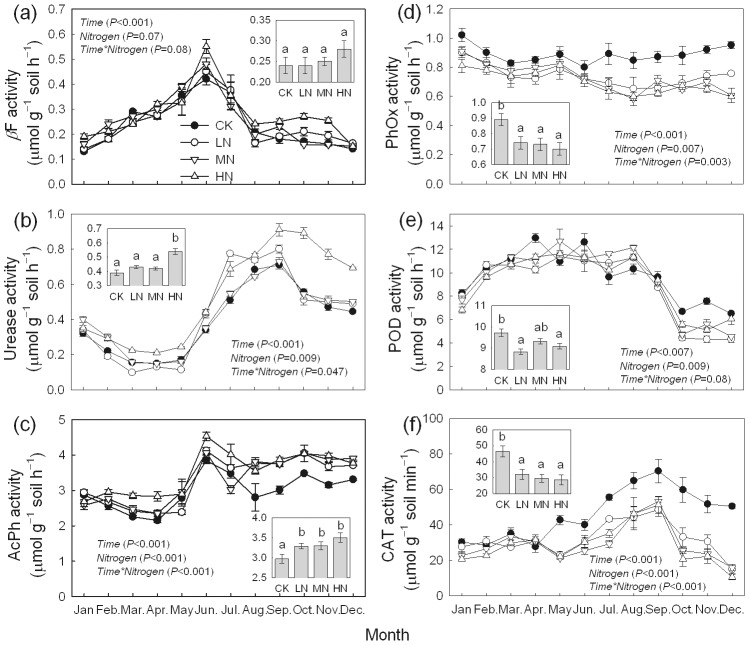
Seasonal variations of soil enzyme activities in a *Bambusa pervariabilis* × *Dendrocalamopsis daii* plantation from January to December 2009 (mean ± SE, *n* = 3). βF: β-Fructofuranosidase; AcPh: acid phosphatase; PhOx: Phenol oxidase; POD: peroxidase; CAT: catalase. Results of repeated measures ANOVAs are shown in text. Different letters indicate significant difference among N addition treatments when nitrogen effect is significant (*P*<0.05, Fisher's least significant difference test). CK, LN, MN and HN are as in Fig. 2.

All six enzyme activities demonstrated significant seasonal variation (P<0.01), and the addition of N changed the seasonal variation significantly (P<0.05) (Fig. 5). The peak period of activity of βF and AcPh occurred in June, and the activity of urease and CAT peaked in September. There was an obvious trough in POD activity from October to December. Results of repeated measures ANOVA tests indicated that N addition significantly increased the activity of urease and AcPh, significantly decreased the activities of PhOx and CAT significantly, and had no significant effect on βF activity. The activity of CAT decreased 31%–38% under N addition treatment, while N addition increased AcPh activity by 11%–18%.

The results of accumulative activities in 1 kg soil in one year are displayed in Table 1. Under N addition treatments, accumulative activities of *β*F and POD increased and decreased respectively, while the differences among treatments were not significant for both enzyme activities. Nitrogen addition significantly increased the urease accumulative activity in the HN treatment. The accumulative activity of AcPh was stimulated, and the accumulative activities of AcPh to all N addition treatments were significantly higher than that of the control. For PhOx and CAT, the accumulative activities were significantly depressed in all the three N addition treatments.

**Table 1:** Results of one-way ANOVAs of accumulative soil enzyme activities in a *Bambusa pervariabilis* × *Dendrocalamopsis daii* plantation (mean ± SE, *n* = 3).

Treatment	βF	urease	AcPh	PhOx	POD	CAT
	mol kg^−1^ yr^−1^	mol kg^−1^ yr^−1^	mol kg^−1^ yr^−1^	mol kg^−1^ yr^−1^	mol kg^−1^ yr^−1^	mol kg^−1^ yr^−1^
Control	2.10±0.16a	3.46±0.13a	26.1±0.6a	7.77±0.37b	85.0±3.0a	24453±1857b
Low-N	2.13±0.16a	3.75±0.12a	28.8±0.6b	6.47±0.35a	77.2±2.7a	16817±1648a
Medium-N	2.22±0.10a	3.65±0.13a	28.9±0.9b	6.38±0.35a	81.6±2.7a	15470±1281a
High-N	2.44±0.16a	4.74±0.15b	30.6±1.1b	6.11±0.31a	79.6±4.7a	15015±1566a

CK, LN, MN and HN denote control (without N added), low nitrogen (50 kg N ha^−1^ year^−1^), medium nitrogen (150 kg N ha^−1^ year^−1^), high nitrogen (300 kg N ha^−1^ year^−1^), respectively; βF  =  β-Fructofuranosidase; AcPh  =  acid phosphatase; PhOx  =  Phenol oxidase; POD  =  peroxidase; CAT  =  catalase. Different letters within the same column indicate significant difference among treatments (one-way ANOVA followed by Fisher's least significant difference test, *α* = 0.05).

## Discussion

Nitrogen and phosphorus are two important elements for ecosystem functioning for both of them commonly limit plant growth [41]. The response of plant production to modern human influences, such as atmospheric deposition and climate change, will be mediated by changes in the availability of these elements [42]. The response of soil nutrients and organic C, especially the active portions, to N addition can reflect the effect of N input on soil C pools of plant and soil. Soil extracellular enzymes mediate the degradation, transformation and mineralization of SOM [15]. In general, hydrolytic enzymes are relevant to decomposition of SOM, and oxidative enzymes relate to the resynthesis of SOM [15]. Therefore, the variations of the two types of enzymes reflect SOM decomposition and humification in soils. In the present study, the addition of N had no significant effect on soil total N concentration, but increased the concentrations of TOC, NH_4_^+^, NO_3_^−^, MBN, MBC, and decreased AP concentration significantly. Two types of hydrolytic enzymes (urease and AcPh) were stimulated significantly, while the three oxidative enzymes were depressed significantly.

Available N in the soil mainly existed in the forms of NH_4_^+^ and NO_3_^−^, which is mainly generated from N mineralization. Urease is a type of enzyme produced by microbes or plants for accessing N. In this study, urease activity was stimulated by N addition, which suggested that the mineralization of organic N may be accelerated by N addition. It was consistent with the results in different forest ecosystems [20]. However, in a tallgrass prairie ecosystem, Ajwa et al. [43] found that N addition inhibited urease activity by approximately 15%. The rates of gross nitrification and denitrification were stimulated by N addition in this bamboo forest. The phenomenon of N addition increasing N transformation was observed in many other systems, such as temperate hardwood forests [44] and exotic annual grasslands [45]. Soil N transformation is a substrate limited process. The nitrification rate depends on the concentration of ammonia, and the nitrate concentration, C availability, as well as oxygen availability [46]. Nitrification and denitrification are main sources of N_2_O emissions from soils [47]. Then, the results suggest that N_2_O emission may be increased in this ecosystem. Actually, Barnard and Leadley [47] conclude in their review that the stimulation of field N_2_O emissions through nitrification and denitrification processes by N additions is clearly shown by global N addition experiments conducted in agricultural and forested ecosystems. The denitrification process might be a mechanism to avoid N imbalance and reduce the negative effects from excess N in forests [48]. The atmospheric N deposition in the experimental area was extremely high compared to the rest of the world [27]. It can be anticipated that the bamboo system in this study and many other similar forest systems in this region would become N saturated systems within decades if the deposition of N continues to increase. Meanwhile, in a bamboo forest (P. amarus) adjacent to the study site, we observed very little N loss through hydrological processes, which indicates that most of the N was lost through denitrification [27]. However, the amount of N lost through denitrification may differ among forest types because of the variability of forest properties and ambient N deposition. Nitrification and denitrification play key roles in regulating soil inorganic N concentration, the production of a potent greenhouse gas N_2_O, and leaching of nitrate [47]. Thus changes in both processes in response to elevated N deposition can influence N stocks and directly feed back to atmospheric and climatic change.

In contrast, the available P in soil decreased significantly after the addition of N in this study. Many previous studies suggested that in N limited systems, N addition increases microbial activity and stimulates the demand for P [18], [20], [43]. Almost all the N addition studies reported the upregulation of the enzymes involved in P turnover [20], [21], [43] and is in accordance with the results of this study. This common phenomenon was revealed by a global meta-analysis, which demonstrated that phosphatase activity was significantly greater in soils supporting symbiotic N_2_-fixing plants because they could afford to invest N in P acquisition [49]. It is well established that phosphate represses the synthesis of phosphatases [50]. In this bamboo ecosystem, although the increased AcPh activity indicates an accelerated rate of transformation of organic P to inorganic P, the elevated microbial activity may have simultaneously accelerated the immobilization of inorganic P and led to the decrease of available P in the soil, and then the higher activity of AcPh. The results in this study combined with previous studies suggest that ecosystems under high levels of N deposition are moving towards being limited or co-limited by P. In fact, many ecosystems around the world are now considered to be co-limited by P [51].

Microorganisms play an important role in nutrient transformation in forest soils. Soil microbial biomass depends on total organic C content, the C stock of the litter, and microbial flora induced in the decomposition of litter and rhizospheric depositions [52]. Similar to this study, N addition increased microbial biomass in a loblolly pine (Pinus taeda) plantation [12], but several other studies have presented contradictory results [53]. There were several possible underlying mechanisms resulting in the phenomenon observed in this study. First, the addition of inorganic N directly provides a vital nutrient for microbial growth. Second, N addition may increase microbial biomass through stimulating the growth and metabolism of fine roots. For example, in our another N addition study, we found fine root biomass and root tissue N concentration increased significantly under N addition [29]. Plant root tissue N concentrations are generally highly correlated with root metabolism rates [54]. The increased microbial biomass in this study suggests elevated potential microbial activity and C turnover rate in this bamboo ecosystem. However, microbial biomass declined 15% on average under N addition on a global scale [55].

Soil oxidative enzymes, especially the lignin-degrading enzymes (PhOx and POD) were closely related to humification [15]. Lignin is a class of three-dimensional acrylic polymers; most of the chemical bonds in which are difficult to hydrolyzed [56]. Compared with polysaccharides and other biopolymers, lignin is highly resistant to decay [57]. There are two kinds of aerobic fungi that break down lignin: white-rot organisms (Basidiomycetes) and soft-rot organisms (Ascomycetes). Degradation carried out by the soft-rot organisms is incomplete, while the white-rot organisms are able to degrade the lignin completely [56]. The production of lignin-degrading enzymes, such as PhOx and POD, by lignin-degrading fungi decreased after inorganic N addition [17]. White-rot organisms did not synthesize lignin-degrading enzymes in the presence of low molecular N-rich compounds (for example, ammonium and amino acids) [56]. In this study, the inhibiting effect of N addition on PhOx and POD can be interpreted as the growth limitation of white-rot organisms caused by soil acidification. The optimum pH values for white-rot and soft-rot organisms are 4.0–5.0 and 6.0–7.5 [15]. Actually, a number of species of white-rot organisms with this property suggests that the lignolytic enzyme production inhibited by high N levels could be a widespread phenomenon [57]. Humus accumulated in the forest soil surface as a result of the N inhibitory effect on lignin decay [58], and the phenomenon would be more obvious when the lignin content in forest litter is higher [16].

We conducted a leaf litter decomposition experiment at the same site during this period. It demonstrated that N addition depressed the leaf litter decomposition through inhibiting the decay of lignin [13], which confirmed the inhibitory effect of N on oxidases activities in this study. Similar to this study, Sinsabaugh et al. [59] found that N deposition decreased oxidase activities significantly in a boreal forest. Analyses of laccase gene abundance and diversity indicated that decreased activity of oxidative enzymes is the result of reduced expression [60]. Freeman et al. [61] proposed that PhOx may act as an “enzymatic latch” and control C storage in many ecosystem types. For example, peatlands, which are considered enzyme-limited systems, have low PhOx activities that contribute to organic matter accumulation [61], [62], while arid ecosystems appear to be substrate-limited systems where high POD and PhOx potentials are believed to limit SOM accumulation [63]. Waldrop et al. [16] conducted a simulated N deposition experiment in several temperate forest ecosystems. They found a significant relationship between changes in PhOx and changes in soil C content across three ecosystems, which indicates that the mechanism of oxidative enzyme activities control soil C storage. The results in this study suggest that more SOM may accumulate in the soil surface through depressing oxidative enzyme activities and inhibiting litter decomposition.

The inhibitory effect of N addition on oxidative enzyme activities is not consistent throughout ecosystem types. Most N addition studies have been conducted in temperate and boreal forests where soil microbial communities are dominated by Basidiomycetes [64]. Grassland soils are dominated by Glomeromycota and Ascomycota [65] and do not show decreased oxidative enzyme activities under N addition treatment [66]. Keeler et al. [18] demonstrated that the negative response of oxidative enzyme activity to N addition is not universal with a long-term N addition experiment conducted in eight different ecosystems. Furthermore, the responses of extracellular enzymes in soils and litter to N addition are not entirely in agreement, due to the differences of internal components, structures and decomposer communities. Saiya-Cork et al. [20] reported that PhOx activity increased in litter but dropped in soil under N addition.

Enzymes themselves are N-rich (protein) so the enzyme production was adjusted by soil N availability [21]. Extracellular enzymes were produced by microbes and plant roots, thus N addition may indirectly affect enzyme activities by affecting enzyme producers. For example, in our previous study conducted in a P. amurus plantation, N addition stimulated aboveground biomass, fine root biomass [29], and the growth of rhizospheric microbes. The results in this study indicate that N addition may play a positive role in plant growth of hybrid bamboo.

Overall, in this hybrid bamboo forest, N addition accelerated nitrification and denitrification rates, increased hydrolytic enzymes activities, soil N availability and microbial biomass. Nitrogen addition stimulated AcPh activity and decreased the soil P availability for the more demands of plants and soil microbes for element P under elevated N addition, suggesting this bamboo system is moving towards being limited by P or co-limited by P and N. The inhibitory effects of N addition on soil oxidative enzyme activities suggest that the expected progressive increases in N deposition may have a potential important effect on forest litter decomposition in such bamboo forests.
